# The Inwardness of Spring Time

**Published:** 1909-04-24

**Authors:** 


### The Hospital

A Journal of

The Medical Sciences and Hospital Administration.

New Series. No. 112, Vol. V. [No. 1184, Vol. XLVI.] SATURDAY, APRIL 24, 1909.

### The Inwardness of Spring Time.

A recent leading article in the *Times* deals suggestively with the influence of spring upon human well-being. The writer observes that poets have taken far too limited a view of this (poetically) pleasant season. True to their trade, they would confine us to an appreciation of the natural beauties which mark the awakening of the year, beauties which we are all ready enough to praise, and perhaps to praise disproportionately after the gloom and discomforts of a long or rigorous winter. But the reverse of the medal is far from pleasing. If we are to believe the leader-writer, there is no doubt that the "germinal period" of the year is notable as a time of prevailing sickness and still more widely prevailing lassitude among civilised races. It is suggested that our vital forces, overtaxed in resisting the adverse influences of winter, undergo a reaction with the appearance of more genial conditions, and leave us victims to the microbic hosts among which it is our fortune to live. Upon this hypothesis he explains both the sicknesses and the lassitude which are credited to springtime, the latter representing the effects of microbic assaults which, though insufficient to produce a definite malady, yet have their expression in a lack of energy and loss of tone. He observes very truly that the micro-organisms responsible for the diseases which run riot in spring are blood-brothers of the larger vegetable world: their growth is profoundly dependent upon temperature, and in a milder air they may well flourish and multiply at a speed impossible under colder skies, behaving in this respect like the rest of the vegetable creation.

There is no doubt, we imagine, that the cold months are more prolific of microbic disease in general than are the warm ones, but it is open to question whether it is just to damn spring-time with greater blame than winter. This is, of course, a matter of opinion, and will probably remain so owing to the unconventionality of our climate. The writer speaks of the "germinal period," and we wonder what exactly he means thereby. Does February, for example, fall within the germinal period? For this year the epidemic of influenza and other microbic ailments reached its height in that month, and has since been steadily declining. When one considers the extraordinary variability of our climate anything like intimate correlation of diseases with seasons becomes profoundly unconvincing. One feels that the argument which credits the diseases of spring to the multiplication of microbes under the influence of warmth is much too narrow, even though one accepts the doubtful premiss that spring is more culpable than winter. The proposition that the cold months are the months of microbic disease *par excellence* is more unassailable, and admits of a fairly simple explanation. The cold months are months of far more intimate human contact than are the warm ones. They are months of indoor existence and of deficient ventilation; for it requires no little force of conviction to live with widely open windows during a hard winter, even though the purse runs to roaring fires on the hearth, and when this happy consummation is out of sight, the longed-for warmth is certain to be sought by conserving the animal heat of the body in a closed room. It is plain, therefore, that once a microbic infection is established in a community under such conditions, the way is paved for its rapid propagation, and this in an increasing scale of virulence, for it is a well-known principle of bacteriology that the virulence of pathogenic organisms is heightened by "passage" through a series of susceptible individuals. It remains to consider the factors which favour the commencement of a microbic descent upon a community thus prepared for its dissemination. The classical experiment of Pasteur upon the susceptibility of chickens to anthrax seems to show conclusively, in spite of modern "fresh air" dogmatists, that exposure to cold is capable of destroying a pre-existing immunity to microbic infection. If this be so, it is easy to believe that an individual already reduced in resistance by a sedentary and confined life, may still further be reduced by exposure to winter air (when occasion takes him out of doors) until the point is reached at which some organism haunting his air-passages finds an outlet for its activities and becomes pathogenic.

The writer in the *Times* draws a somewhat ingenuous deduction from the points he has advanced. It is that since tastes, when not artificially vitiated, are to be accepted as indications of the wants of the system, a period of disinclination for work shows the need for a corresponding relaxation of effort. He observes, truly enough, that the literary man, who perseveres with his work in spite of a consciousness of being "out of form," often finds that the task thus laboriously completed is quite unsatisfactory and requires to be done over again. "Nature often speaks eloquently enough if we will but listen to her; and her warnings can seldom be neglected with impunity." We accept the hint, but it is surely too much to say that the lassitude he claims for spring-time and its microbes is the general cause of loss of "form." Variation in "form" is most easily gauged in games of skill requiring co-ordination of hand and eye, like billiards, and nothing is more singular than the rapidity with which "form" is developed and lost, perhaps in the course of a single game. Our intellectual activity may pass through analogous phases, though they are not so readily identified, but it is incredible that these variations depend upon the passing attentions of microbes.

